# Choked Vein: Unusual Etiology of Extensive Deep Vein Thrombosis

**DOI:** 10.7759/cureus.8292

**Published:** 2020-05-26

**Authors:** Salem Gaballa, Kyaw M Hlaing, Nadine Bos, Gretchen Junko, Abdullah Alcharif

**Affiliations:** 1 Internal Medicine, LewisGale Medical Center, Salem, USA; 2 Internal Medicine, LewisGale Medical Center, Roanoke, USA

**Keywords:** deep vein thrombosis, inferior vena cava atresia, heterozygous factor v leiden mutation

## Abstract

Deep venous thrombosis (DVT) is a commonly encountered diagnosis in clinical practice with a variety of established risk factors. Inferior vena cava atresia (IVCA) is a rare vascular anomaly, but an established risk factor, associated with DVT, found in approximately 5% of cases of unprovoked lower extremity DVT in young adults. Patients who develop DVT are at high risk of long-term complications, including DVT recurrence and post-thrombotic syndrome. Thirty percent of inferior vena cava (IVC) anomalies are associated with hypercoagulable conditions in the younger population, Therefore, a hypercoagulable workup is beneficial in this population. We report a rare case of a 31-year-old male who presented with an extensive DVT of bilateral lower extremities secondary to IVC atresia. The treatment of choice for IVC atresia associated with extensive DVT is catheter-directed thrombolysis (CDT), endovascular IVC reconstruction with nitinol stent, and long-term anticoagulation.

## Introduction

Inferior vena cava atresia (IVCA) is a rare vascular anomaly associated with deep venous thrombosis (DVT), found in approximately 5% of cases of unprovoked lower extremity DVT in the younger population (<30 years) [1]. IVCA- associated DVT is characterized by happening in the early thirties, predominantly male, often with a history of major physical exertion, and presents with extensive or bilateral DVTs. Thirty percent of inferior vena cava (IVC) anomalies are associated with hypercoagulable conditions in the younger population. A hypercoagulable workup is beneficial in this population [2].

## Case presentation

A healthy 31-year-old male with no past medical history presented with low back pain associated with new-onset right lower extremity swelling and numbness. He stated that over the past month, he drove to Canada and took a 10-hour flight to Moldova. Upon return to the US, he went to his primary care provider for lower back pain and was given a prescription for oral diclofenac. Due to persistent severe back pain, he presented to the emergency department and was evaluated. Pertinent physical findings were right lower extremity (RLE) erythema, warmth, and moderate pitting edema along with a positive Homan’s sign and bilateral lower back pain. His back was tender with light palpation rated 8/10.

**Figure 1:**
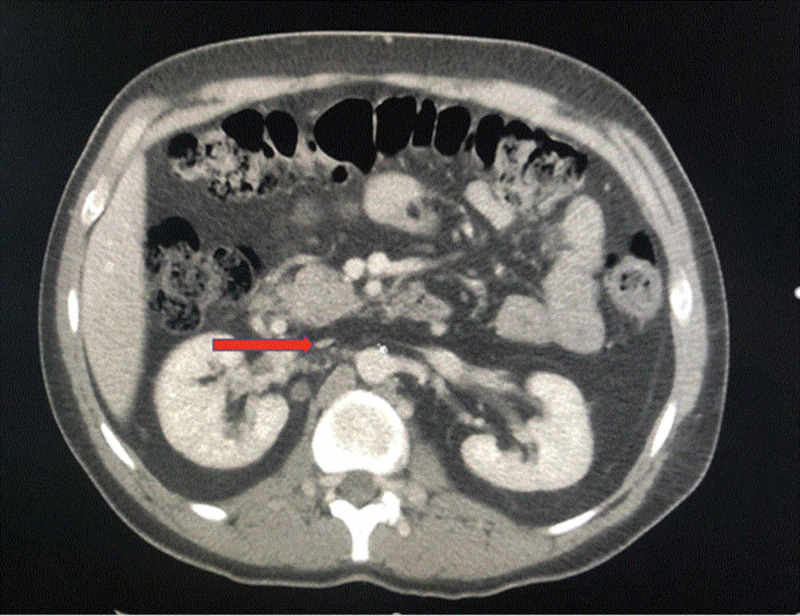
Computed tomography of the abdomen and pelvis with IV contrast, in an axial view, shows an atretic infrarenal inferior vena cava (red arrow)

**Figure 2:**
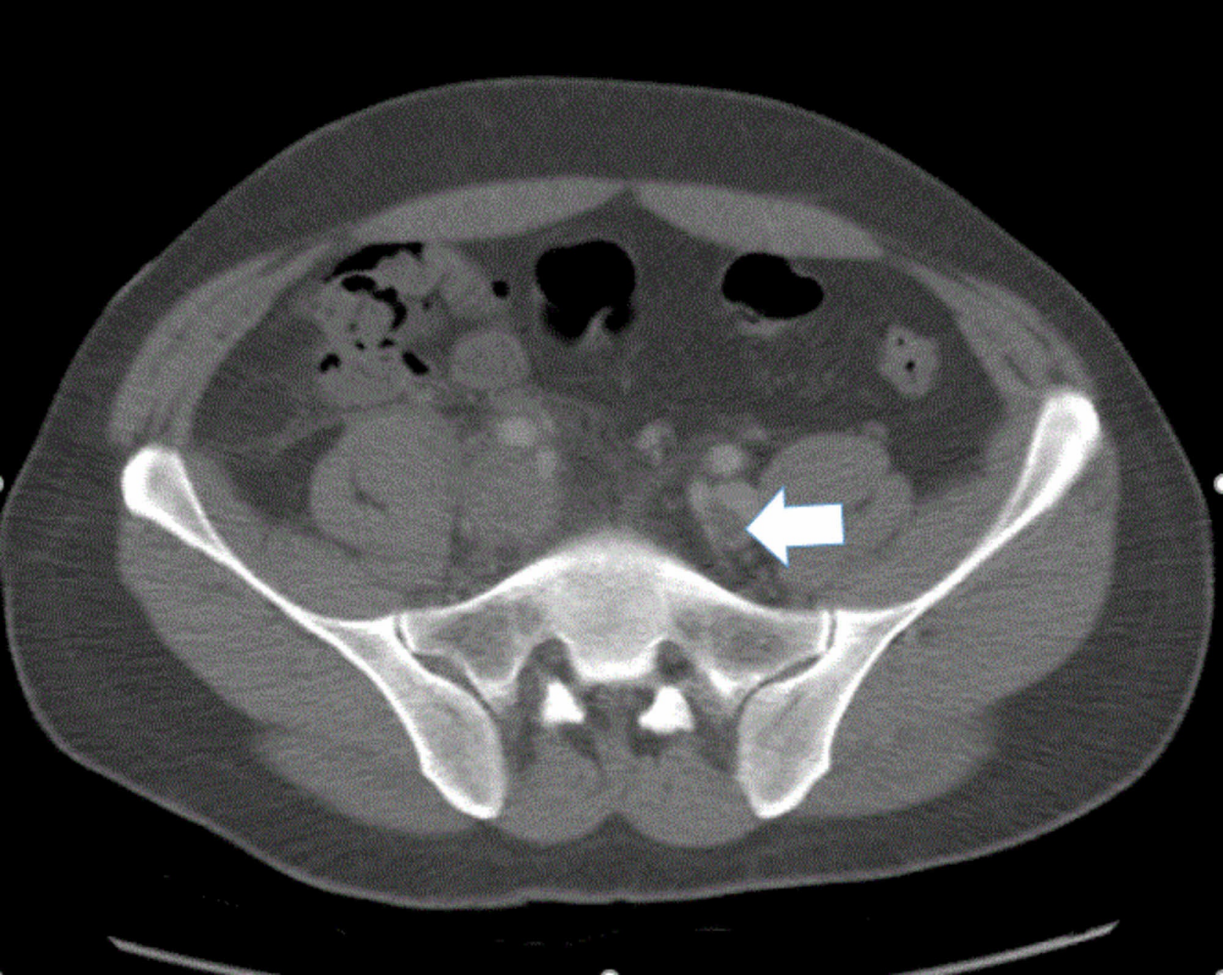
Computed tomography of the abdomen and pelvis with IV contrast, in an axial view, shows left external iliac vein thrombosis (white arrow)

**Figure 3:**
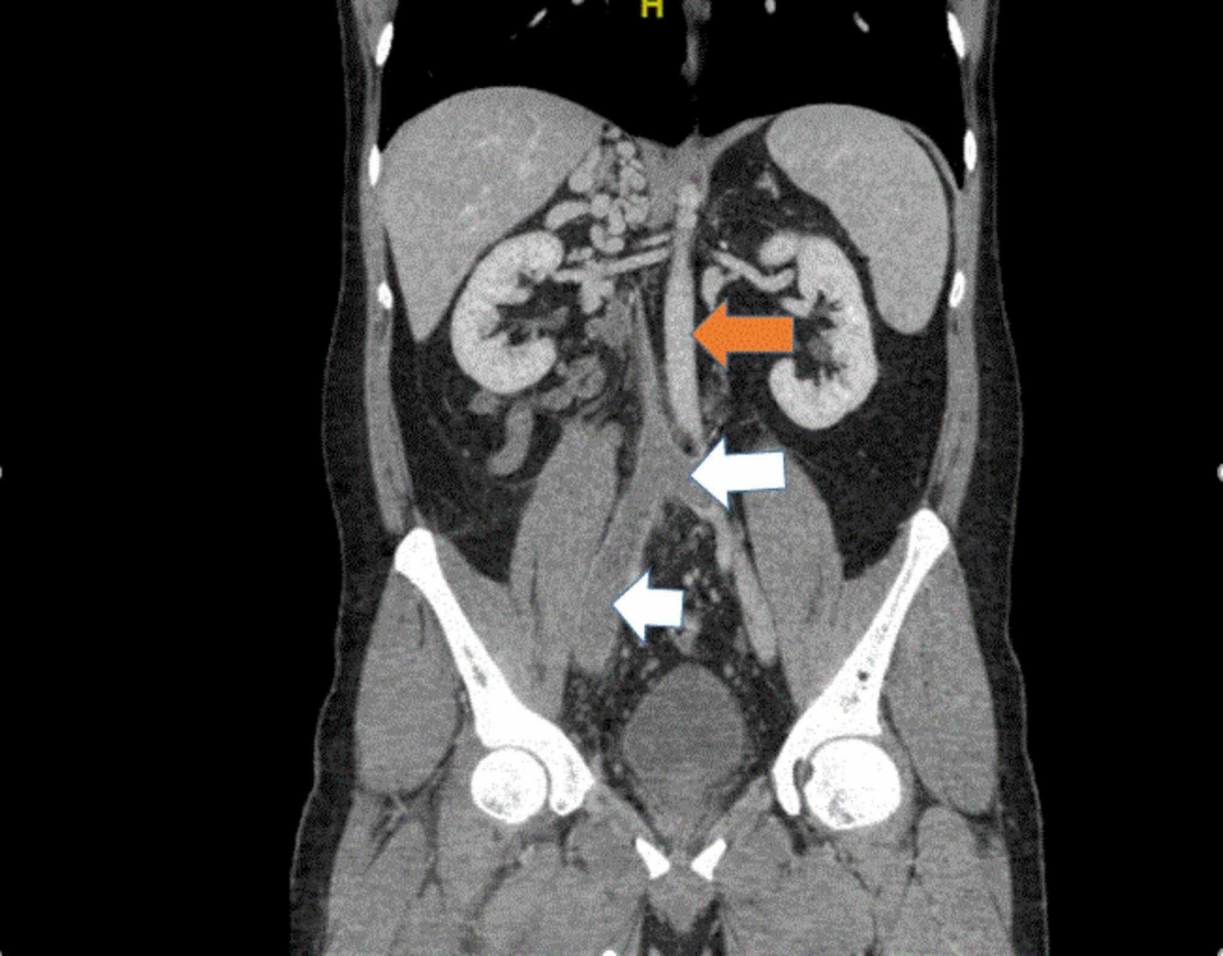
Computed tomography of the abdomen and pelvis with IV contrast, in coronal view, shows extensive right and left common iliac vein thrombosis (white arrow) and normal aorta (orange arrow)

**Figure 4:**
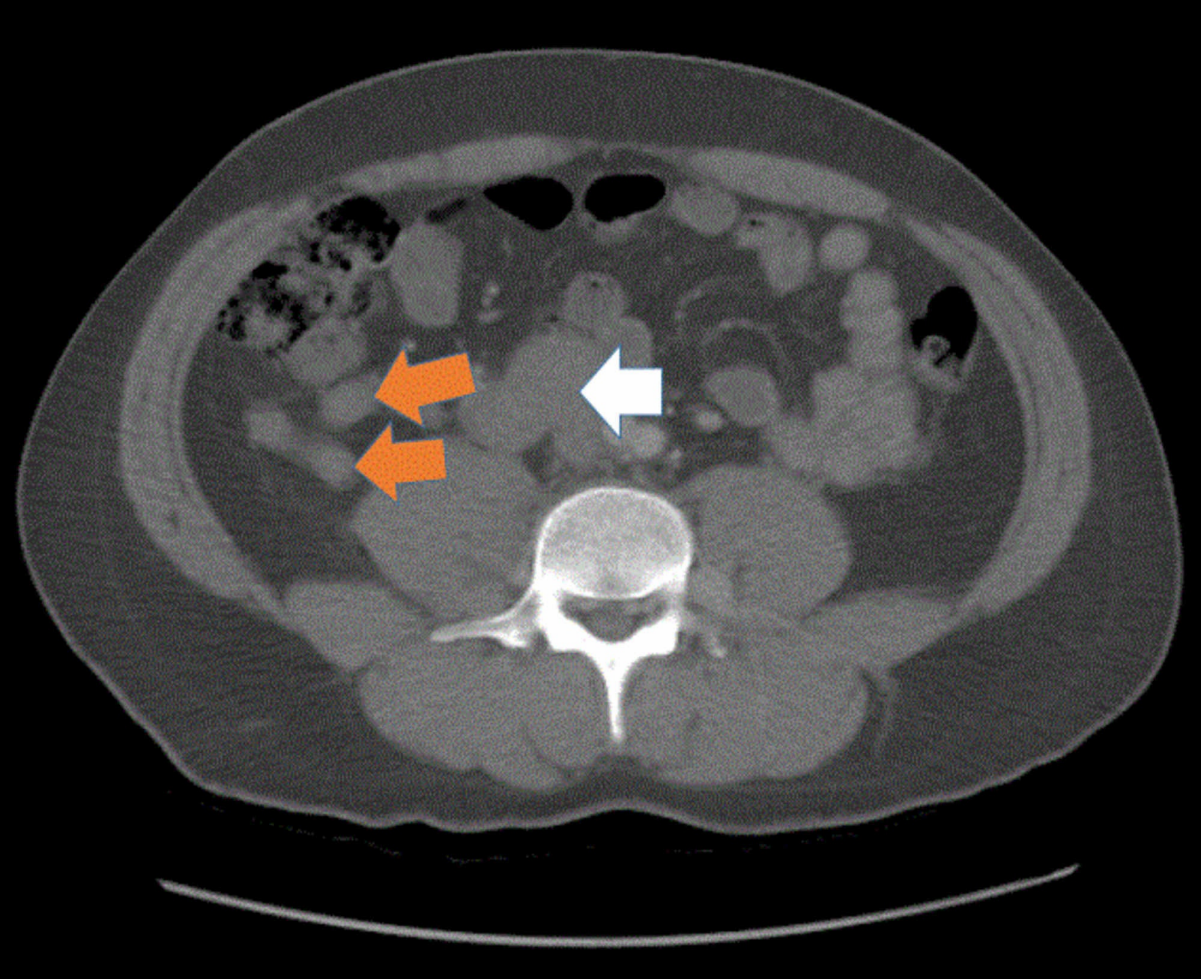
Computed tomography of the abdomen and pelvis with IV contrast, in an axial view, shows large retroperitoneal venous varix (white arrow) and small retroperitoneal collaterals (orange arrows)

**Figure 5:**
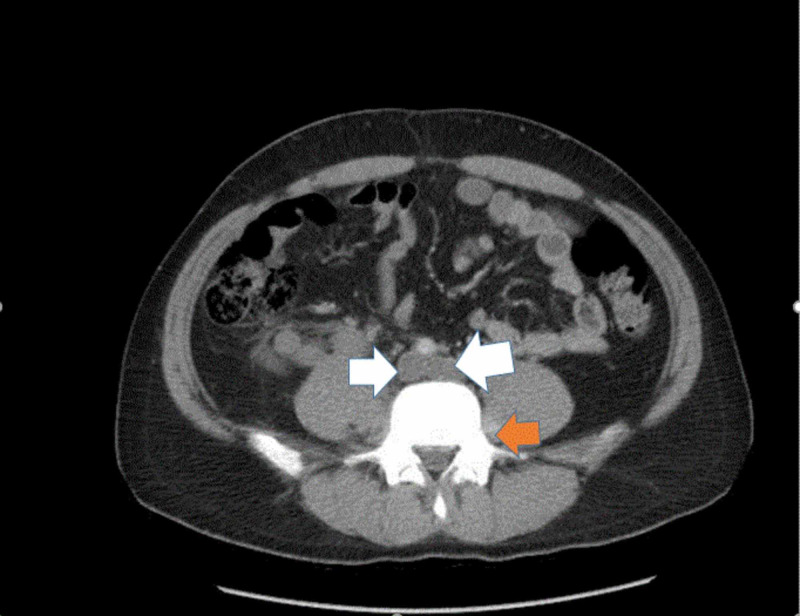
Computed tomography of the abdomen and pelvis with IV contrast, in an axial view, shows left lumbar vein thrombosis (orange arrow) and azygos and hemiazygos vein thrombosis (white arrows)

Computed tomography angiography (CTA) of the chest/abdomen/pelvis showed an atretic infrarenal IVC, right common and external iliac vein thrombosis, and left common iliac vein thrombosis to its bifurcation, large thrombosed collaterals in the right retroperitoneum, and left lumbar vein, and azygous/hemizygous vein thrombosis (Figures 1-5). There was no evidence of pulmonary embolism. Coagulation studies showed a normal prothrombin time (PT) and international normalized ratio (INR) but elevated activated partial thromboplastin time (APPT) (46 sec) and fibrinogen (503.7 mg/dL). Complete blood count (CBC) showed a hemoglobin of 11.1 with no thrombocytosis or erythrocytosis. Thrombophilia workup was positive for heterozygous factor V Leiden mutation but negative for prothrombin gene mutation, hyperhomocysteinemia, and Jak2 kinase mutation.

**Figure 6:**
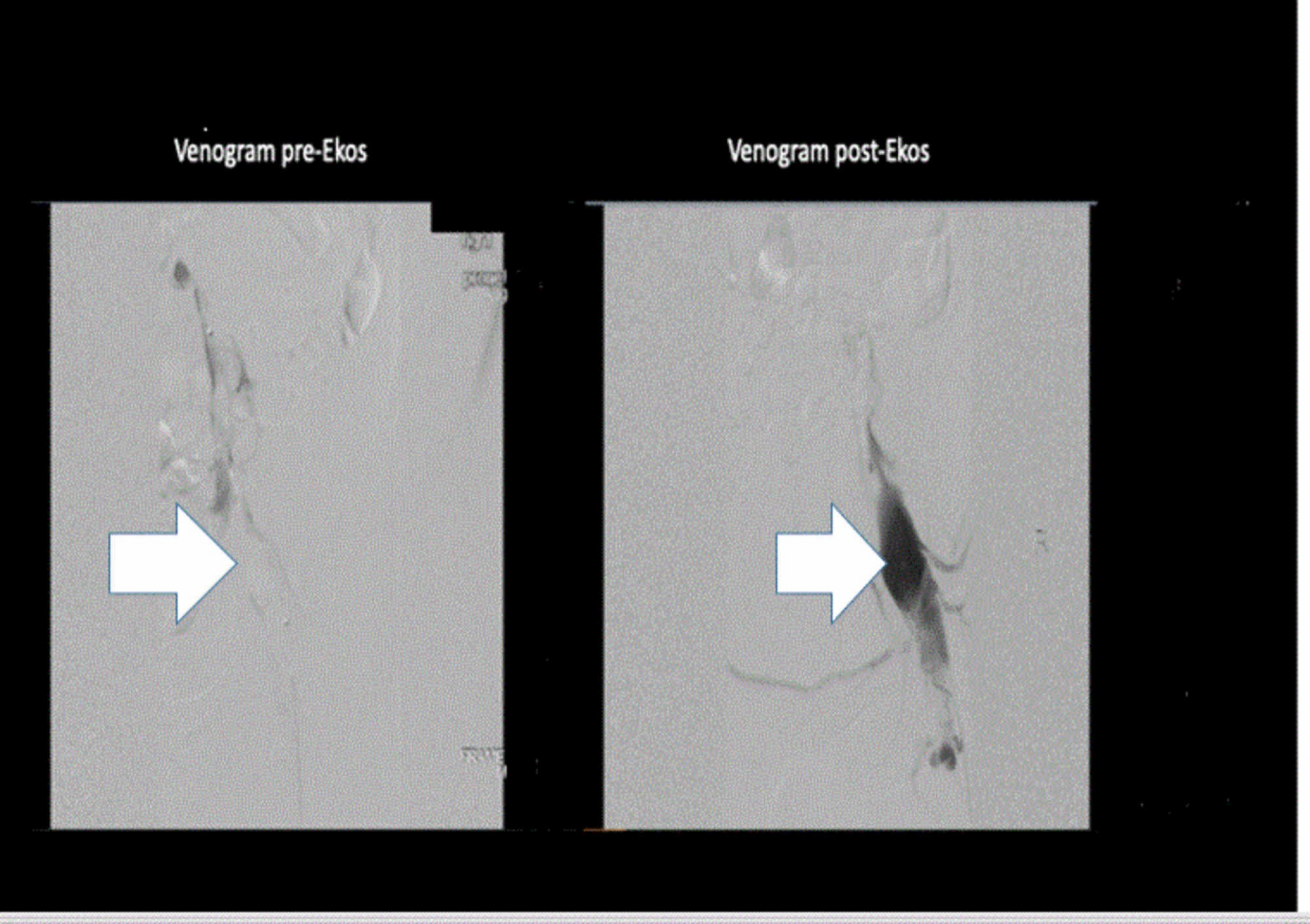
Pre-Ekos venogram shows extensive acute occlusive thrombus of the right femoral vein at the level of the femoral head (white arrow). Post-Ekos venogram shows almost a complete resolution of the thrombus and the return of the blood flow (white arrow)

A heparin bolus followed by a drip was administered and the patient was seen by both interventional radiology and vascular surgery. Right lower extremity venogram showed extensive acute occlusive thrombus of the right femoral vein at the level of the femoral head (Figure 6). Interventional radiology advanced an EkoSonic 5.4F 135 cm ultrasound accelerated thrombolysis catheter to the level of the clot. Alteplase and heparin were run through the catheter lumen to the clot. Ultrasound was initiated and the catheter was left in place for 24 hours. After 24 hours, the catheter was removed. Post-thrombolysis catheterization venography showed almost complete resolution of the acute right femoral vein thrombus and the return of blood flow (Figure 6). IVC reconstruction from an inferior approach was unsuccessful as the catheter preferentially entered the larger IVC collaterals rather than the atretic portion. Recommendations from the interventional radiologist included lifelong anticoagulation and consideration of endovascular iliocaval venous reconstruction from a superior approach using bilateral “kissing” stents at the level of IVC atresia/obstruction.

## Discussion

IVCA or agenesis is often congenital or acquired because of either embryonic dysgenesis or thrombosis during the intrauterine period. During embryogenesis period, the IVC is formed by the fusion of three sets of paired veins (posterior cardinal, subcardinal, and supracardinal veins). The failure of these veins to fuse into venous structure results in the anomaly of IVC. The presentation of IVC atresia as an extensive deep vein thrombosis with thrombosis or hypertrophy of collateral vessels is a rare clinical form. It is presumed that the collaterals developed before deep vein thrombosis are unable to cope with the increased blood flow, thereby generating venous stasis and recurrent deep vein thrombosis. The presence of thrombus induces an inflammatory response, which in return causes valvular scarring and incompetence, therefore promoting venous reflux and chronic venous hypertension. All of these changes would cause limb swelling, stasis dermatitis, and recurrent DVT [3]. Pulmonary embolism is an uncommon finding with IVCA because the emboli get trapped in the azygos, and hemiazygos system preventing it from reaching the pulmonary circulation [4].

Early intervention is essential to reduce the risk of deep vein thrombosis recurrence and the occurrence of post-thrombotic syndrome. The characteristic of IVC atresia-associated DVT is occurrence before the fourth decade of life, usually male predominance, and extensive or bilateral lower extremities DVT [5]. The DVT most frequently involves the distal IVC, common, internal, and external iliac and femoral veins.

The duration of anticoagulation is debatable. However, in the absence of other risk factors of DVT, a short duration of three to six months is sufficient. In the presence of other risk factors as thrombophilia, chronic immobilization, or malignancy, lifelong anticoagulation is generally indicated [5,6]. Gayer et al. suggest a higher incidence of recurrent DVT with co-existing IVC anomalies and thrombophilia [7]. Therefore, a hypercoagulable workup as thrombophilia screening should be performed in all patients found to have IVC anomalies presenting with DVT.

Ultrasound and venography are excellent tools in the identification of deep vein thrombosis, but they frequently miss the diagnosis of IVCA. CT or MRI with contrast is more effective in identifying IVC anomalies and is recommended if there is a history of unprovoked deep vein thrombosis in younger patients [7].

Endovascular management with catheter-directed thrombolysis (CDT) is safe and effective in reducing thrombus burden and restoring venous patency. Therefore, decreasing the risk of post-thrombotic syndrome and chronic venous insufficiency. Bush et al. suggested that catheter-directed methods are considered superior to systemic thrombolysis due to the high risk of bleeding [8]. Further, the authors suggested that pharmacomechanical catheter-directed thrombolysis (PCDT), which refers to the combination of mechanical thrombectomy and CDT, has been shown to decrease the thrombus burden, the incidence of recurrent DVT, and the incidence of the post-thrombotic syndrome [8-10].

In a case report by Dougherty et al., surgical option is an alternative to endovascular therapy in cases of extensive iliofemoral DVT associated with IVC anomalies. Approaches typically involve reconstruction of the absent IVC via placement of bypass grafts, thus permitting a return of venous outflow and resulting in significant symptomatic relief [11].

## Conclusions

IVCA can be congenital or acquired due to either embryonic dysgenesis or thrombosis during the intrauterine or perinatal period. Deep vein thrombosis of the IVC veins and femoral veins may be associated with congenital anomalies of the IVC, specifically in young patients with bilateral deep vein thrombosis. A thorough investigation of thrombophilia markers may also be useful to complete the evaluation of these patients. The treatment of choice for IVCA with symptomatic heterozygous factor V Leiden are PCDT, endovascular IVC reconstruction, and long-term anticoagulation.

## References

[1] Shalini K, Smith AG, Dhillon RK (2015). Incidental finding of inferior vena cava atresia presenting with deep venous thrombosis following physical exertion. Case Rep Emerg Med.

[2] Rogers A, Moloney MA, O'Donnell DH, Sheehan S, Brophy DP (2010). Deep venous thrombosis in a patient with atresia of the infrarenal inferior vena cava. J Vasc Interv Radiol.

[3] Prandoni P, Noventa F, Ghirarduzzi A (2007). The risk of recurrent venous thromboembolism after discontinuing anticoagulation in patients with acute proximal deep vein thrombosis or pulmonary embolism. A prospective cohort study in 1626 patients. Haematologica.

[4] Muscianese L, Seese RR, Graham W, Williams JH (2015). Congenital atresia of the inferior vena cava and antithrombin III deficiency in a young adult: compounding risk factors for deep vein thrombosis. BMJ Case Rep.

[5] Lambert M, Marboeuf P, Midulla M (2010). Inferior vena cava agenesis and deep vein thrombosis: 10 patients and review of the literature. Vasc Med.

[6] Moore MR, Hopkins JH Jr (2018). Congenital inferior vena cava atresia presenting as extensive thrombosis in adulthood. Am Acad Pediatr.

[7] Gayer G, Luboshitz J, Hertz M (2003). Congenital anomalies of the inferior vena cava revealed on CT in patients with deep vein thrombosis. AJR Am J Roentgenol.

[8] Bush RL, Lin PH, Bates JT, Mureebe L, Zhou W, Lumsden AB (2004). Pharmacomechanical thrombectomy for treatment of symptomatic lower extremity deep venous thrombosis: safety and feasibility study. J Vasc Surg.

[9] Kearon C, Kahn SR, Agnelli G, Goldhaber S, Raskob GE, Comerota AJ (2008). Antithrombotic therapy for venous thromboembolic disease. Chest.

[10] Garg K, Cayne N, Jacobowitz G (2011). Mechanical and pharmacologic catheter-directed thrombolysis treatment of severe, symptomatic, bilateral deep vein thrombosis with congenital absence of the inferior vena cava. J Vasc Surg.

[11] Dougherty MJ, Calligaro KD, DeLaurentis DA (1996). Congenitally absent inferior vena cava presenting in adulthood with venous stasis and ulceration: a surgically treated case. J Vasc Surg.

